# Herpes Infections in Suspected Cases of Yellow Fever in the Democratic Republic of the Congo

**DOI:** 10.3390/medicina57090871

**Published:** 2021-08-25

**Authors:** Sheila Makiala-Mandanda, Jessica L. Abbate, Elisabeth Pukuta-Simbu, Steve Ahuka-Mundeke, Jean-Jacques Muyembe-Tamfum, Eric M. Leroy, Pierre Becquart

**Affiliations:** 1 Centre International de Recherches Médicales de Franceville (CIRMF), Franceville BP 769, Gabon; 2 Département de Microbiologie, Cliniques Universitaires de Kinshasa (CUK), Kinshasa BP 127, Democratic Republic of the Congo; amstev04@yahoo.fr (S.A.-M.); jjmuyembet@gmail.com (J.-J.M.-T.); 3 Unité Mixte de Recherche MIVEGEC, Institut de Recherche pour le Développement, UMR IRD/CNRS/Université de Montpellier, 34394 Montpellier, France; jessie.abbate@gmail.com (J.L.A.); eric.leroy@ird.fr (E.M.L.); 4 Unité Mixte Internationale UMMISCO, Institut de Recherche pour le Développement, UMI IRD/Sorbonne Université, 93140 Bondy, France; 5 Institut National de Recherche Biomédicale (INRB), Kinshasa BP 1197, Democratic Republic of the Congo; elisepukuta@gmail.com

**Keywords:** herpes, yellow fever, Democratic Republic of the Congo

## Abstract

In the battle to quickly identify potential yellow fever arbovirus outbreaks in the Democratic Republic of the Congo, active syndromic surveillance of acute febrile jaundice patients across the country is a powerful tool. However, patients who test negative for yellow fever virus infection are too often left without a diagnosis. By retroactively screening samples for other potential viral infections, we can both try to find sources of patient disease and gain information on how commonly they may occur and co-occur. Several human arboviruses have previously been identified, but there remain many other viral families that could be responsible for acute febrile jaundice. Here, we assessed the prevalence of human herpes viruses (HHVs) in these acute febrile jaundice disease samples. Total viral DNA was extracted from serum of 451 patients with acute febrile jaundice. We used real-time quantitative PCR to test all specimens for cytomegalovirus (CMV), herpes simplex virus (HSV), human herpes virus type 6 (HHV-6) and varicella-zoster virus (VZV). We found 21.3% had active HHV replication (13.1%, 2.4%, 6.2% and 2.4% were positive for CMV, HSV, HHV-6 and VZV, respectively), and that nearly half (45.8%) of these infections were characterized by co-infection either among HHVs or between HHVs and other viral infection, sometimes associated with acute febrile jaundice previously identified. Our results show that the role of HHV primary infection or reactivation in contributing to acute febrile jaundice disease identified through the yellow fever surveillance program should be routinely considered in diagnosing these patients.

## 1. Introduction

Due to the severity and the high risk of re-emergence of yellow fever, a disease caused by an arthropod-borne virus, the Democratic Republic of the Congo (DRC) has implemented an active syndromic surveillance program throughout the country since 2003 based on the involvement of healthcare structures located in its health areas. All suspected clinical cases compatible with Yellow fever disease, including fever and jaundice, were notified to the Ministry of Health and rapidly tested for Yellow fever infection. A total of 652 suspected YF cases were detected through the program, of which only less than 5% tested positive for YFV. In order to investigate other causative pathogens, we previously investigated other viral pathogens, sometimes associated with acute febrile jaundice, retrospectively in available samples [1,2]. These target viruses included two transmitted by arthropods (thus known as arboviruses: Chikungunya virus and dengue virus), as well as hepatitis (A, B, C and E) virus infections which are specifically characterized by hepatic disease and which can occur at high frequencies in the study population. We found 3.5% (*n* = 16/453), 0.4% (*n* = 2/453), 16.7% (72/432), 22.3% (*n* = 105/470), 2.3% (*n* = 9/379) and 10.4% (*n* = 38/365) tested positive for Chikungunya virus, Dengue virus, HAV, HBV, HCV and HEV, respectively. Though these investigations associated 224 suspected yellow fever cases with a potential causative pathogen, many remained unidentified. Here, we investigated four human herpes viruses (HHVs) known sometimes to cause acute jaundice and which infect African populations at high prevalence: herpes simplex virus (HSV1 & HSV2), varicella-zoster virus (VZV), cytomegalovirus (CMV), and human herpes virus type 6 (HHV-6) [3,4]. Indeed, the seroprevalence of HSV1, HSV2, and CMV are estimated to be 87%, 31%, and 82%, respectively [5,6,7,8]. The seroprevalence of VZV is variable across countries and studies in Africa (0% to 90%) [9]. Few data exist on the prevalence of HHV-6, but it can reach 100% in many regions of the world [10]. As these viruses typically establish a life-long persistent infection within specific tissues, such as ganglia of the nervous system (HSV, VZV) and lymphoid tissue (CMV, HHV-6) [7], after primary infection, they undergo periods of dormancy during which viral replication and shedding is not observable. A resurgence of viral replication can occur at any time, and may be more likely following infection by a subsequent pathogen. Such co-infections could potentially be responsible for the clinical signs observed in these suspected yellow fever cases. Clinical manifestations are also diverse: HSV-1 causes blisters on the lips and HSV-2 is associated with similar blisters or sores on the genitals, CMV induces pneumonia and meningitis, VZV is responsible for Varicella (chickenpox) or herpes zoster (shingles) after reactivation, and HHV-6 is the cause of the common childhood illness *exanthema subitum* (also known as roseola infantum) [11,12]. 

## 2. Materials and Methods

### 2.1. Clinical Data

Yellow fever surveillance is an operational surveillance program based on a case definition of the infection. Suspected cases of yellow fever are defined as patients with an acute onset of fever followed by jaundice within two weeks of the first symptoms, and that are negative for malaria by thick blood smear or fail to respond to appropriate anti-malarial treatment. Other clinical signs are generally not recorded. The data collection form only includes demographic data such as age, gender, date of collection and health zone of origin [1,2].

### 2.2. Laboratory Investigations

Total viral DNA was extracted from 200 µL of serum and eluted in 120 µL of elution buffer using the EZ1^®^ Advanced XL Biorobot (Qiagen, Valencia, CA, USA) as described previously [1,2].

We used real-time quantitative PCR with previously published primers and probes to test all specimens for CMV, HSV, HHV-6 and VZV [13,14,15]. A total volume of 25 μL was used, with 0.2 µM of each primer and probe, 12.5 μL of TaqMan Universal MasterMix (Applied Biosystems, Branchburg, NJ, USA) and 5 μL of template DNA for HSV, CMV, HHV6 and VZV assays. All reactions were performed on the Applied Biosystems 7500 Real-Time PCR System, using the following conditions: 2 min at 50 °C, 10 min at 95 °C, 45 cycles of 15 s at 95 °C, and 1 min at 60 °C, according to the manufacturer’s protocol.

### 2.3. Statistical Methods

All statistical analyses were performed in the R statistical software environment (version 4.0.5), and statistical significance was evaluated at *p* < 0.05. Differences in HHV infection prevalence (at least one HHV detected among those sampled) between sexes and age (broken down into 3 classes: 0–15 year-olds, 16–40 year-olds, and over 40 years old) were tested using chi-square tests (using *stats* function ‘chi.test’); *p*-values calculated on 1 and 2 degrees of freedom, respectively. Co-infections were evaluated where possible from the 451 samples for which the presence of all target HHVs were tested, and represent only a minimum number of co-infections with other target hepatitis virus and arbovirus infections, as complete data for these were not available for all 451 samples. The influence of sex, age (in years), and their interaction on relative risk of co-infection (mono-HHV infection vs. HHV + co-infection) was tested using a logistic regression (function ‘glm’ in the *stats* package with binomial logit link specified) followed by analysis of deviance (function ‘drop1′ with chi-square statistic, equivalent to likelihood ratio tests) using the 287 samples from which data were available for all target viruses. The interaction term was removed from the model when the initial test revealed that any interaction was not statistically significant (*χ*^2^= 0.077, df = 1, *p* = 0.78).

## 3. Results

We detected DNA for 109 human herpes virus infections in 96 of the 451 samples collected from January 2003 to January 2012 (Table 1). 

Among these herpes virus positive samples, 59 (13.1%), 11 (2.4%), 28 (6.2%) and 11 (2.4%) were positive for CMV, HSV, HHV-6 and VZV, respectively. HHVs were detected in samples without significant differences between sex (*χ*^2^ < 0.01, df = 1, *p* = 0.99) and age groups (*χ*^2^ = 2.20, df = 1, *p* = 0.33) (Table 1A). Interestingly, we reported a total of 44 co-infections, corresponding to 45.8% of the 96 HHV-infected individuals, including 12 (12.5% of 96) with multiple HHV infections. These multiple HHV infections included 1 CMV/VZV, 1 HHV-6/VZV, 2 CMV/HHV-6, 3 HSV/HHV-6, 4 CMV/HSV, and 1 CMV/HSV/HHV-6 (Table 1B). Further breakdown showed that 7 (7.3%) samples were co-infected by two or more HHV, 32 (33.3%) samples co-infected with previously identified hepatitis viruses (HAV, *n* = 12; HBV, *n* = 18; HCV, *n* = 3; and HEV, *n* = 6) or arboviruses (chikungunya, *n* = 2 and dengue virus, *n* = 3), and 5 (5.2%) infected by both multiple herpes viruses and at least one additional target virus (Table 1B). The risk of co-infection was also not significantly impacted by sex (*χ*^2^ = 2.50, df = 1, *p* = 0.52) nor age (*χ*^2^ = 0.42, df = 1, *p* = 0.11).

## 4. Discussion

This study shows that many HHVs were detectable in the blood of acute febrile jaundice patients, as 21.3% (96/451) had a detectable virus for at least one HHV tested. This frequency is most certainly underestimated because detection of viral DNA in blood does not necessarily reflect the amount of virus produced by active infection of distant lymphoid tissue and organs (e.g., HHV6), and viral DNA could be highly fragmented making it difficult to detect by PCR (e.g., CMV) [16,17]. Nearly half of these HHV infections co-occurred with other hepatitis viruses or arboviruses previously identified. An unexpected observation was the detection of viral DNA of two HHVs in the blood in 6.4% of patients. This dual HHV infection, rarely reported in the literature, could be an increased risk factor for HHV disease and the presence of concurrent HHV DNA in the plasma could be an unfavorable prognostic factor [18].

The origin of HHV viral DNA in serum can be a primary infection but is more likely a reactivation of latent virus induced by co-infections highlighted in our study, particularly for viruses such as CMV which are acquired at high seroprevalence rates in childhood. The specific mechanisms for reactivation of HHV are not well known and few data exist to our knowledge on the clinical impacts of co-infection with HHV and hepatitis viruses or arboviruses [19]. The majority of hepatitis viruses establish chronic infections in which constant replication takes place and may impact the immune response which could be responsible for a reactivation of HHV [20,21]. Similarly, acute infection caused by an arbovirus may induce a weakening of the immune response which can enhance replication of persistent viral infections otherwise kept under control [22]. Taken together, co-infection by these viruses may explain the severe symptoms of acute jaundice observed in some patients. However, a large number of patients remain undiagnosed, and the viruses we have so far investigated may be present without explaining the symptoms. Indeed, other pathogens may cause these symptoms. First, we do not have data concerning HIV or if patients were immunosuppressed, which could be an aggravating factor. We have also not looked for bacterial (typhoid, typhus, borreliosis, leptospirosis) nor parasitic infections (malaria, toxoplasmosis, schistosomiasis), which are very common in rural areas with poor sanitary conditions and may induce jaundice [23,24]. We also did not have the resources to test for Epstein-Barr Virus, another HHV that is known to cause hepatic dysfunction [25].

## 5. Conclusions

Diagnosis of acute febrile jaundice remains a challenge in yellow fever surveillance. Our study indicates active herpes virus infection in 21.3% of acute febrile jaundice patients, with nearly half of the infections characterized by the co-detection of multiple active viruses, each known sometimes to produce acute febrile jaundice symptoms These multiple infections may partly explain symptoms compatible with yellow fever disease. These findings should therefore be systematically taken into account in the therapeutic management and the delivery of appropriate palliative care to patients exhibiting yellow fever-like disease. Future studies should address the prevalence of HHVs and co-infections in the general population, in order to fully understand the implication of our results.

## Figures and Tables

**Table 1:** (**A**) Occurrence and demographic characteristics of HHVs detected in patients suspected of yellow fever in the DRC. (**B**) Occurrence and demographic characteristics of mono- and co-detection of HHV and other target viruses.

**(A)**	**CMV**	**HHV-6**	**HSV**	**VZV**	Total* HHV+	*p* Value **
**Occurrence** (*n* = 451)	59 (13.1%)	28 (6.2%)	11 (2.4%)	11 (2.4%)	96 (21.3%)	
**Sex**						0.99
**Male** (*n* = 237)	31 (52.5%)	15 (53.6%)	7 (63.6%)	5 (45.5%)	51 (53.1%)	
**Female** (*n* = 214)	28 (47.5%)	13 (46.4%)	4 (36.4%)	6 (54.5%)	45 (46.9%)	
**Age group, year** (%)						0.33
**0–15** (*n* = 190)	27 (45.7%)	15 (53.6%)	4 (36.4%)	4 (36.4%)	45 (46.9%)	
**16–40** (*n* = 180)	22 (37.3%)	6 (21.4%)	4 (36.4%)	4 (36.4%)	32 (33.3%)	
**> 40** (*n* = 81)	10 (17.0%)	7 (25.0%)	3 (27.2%)	3 (27.2%)	19 (19.8%)	
**(B)**	**Mono-Detection Single HHV**	Co-Detection***				
		With Other HHV	With Other Target Virus (Arbovirus Or Hepatitis Virus)		With Other HHV and Other Target Virus	
Occurrence (*n* = 96)	52 (54.2%)	7 (7.3%)	32 (33.3%)		5 (5.2%)	
Sex ratio (M:F)	0.5 (27:25)	0.6 (4:3)	0.6 (18:14)		0.4 (2:3)	
Median age (Range)	18.5 (1–62)	22 (0–55)	15.5 (1–70)		29 (2–67)	
Total (All target viruses tested) *n* = 287	32 (57.1%)	6 (10.7%)	16 (28.6%)		2 (3.6%)	
Sex ratio (M:F)	0.6 (18:14)	0.5 (3:3)	0.5 (8:8)		0 (0:2)	
Median age (Range)	11 (1–62)	16.5 (0–55)	30 (1–70)		30 (13–47)	

* total number of samples positive for at least one HHV; ** statistical significance was evaluated using likelihood ratio tests; *** Co-Detection categories are mutually exclusive.
